# Epidemic of measles following the nationwide mass immunization campaign

**DOI:** 10.1186/1471-2334-13-139

**Published:** 2013-03-18

**Authors:** Jie Gao, Enfu Chen, Zhigang Wang, Jichuan Shen, Hanqing He, Huilai Ma, Guang Zeng, Bao-Ping Zhu

**Affiliations:** 1 Jing’an District Center for Disease Control and Prevention, Shanghai City, People’s Republic of China; 2 Chinese Field Epidemiology Training Program, Chinese Center for Disease Control and Prevention, Beijing, People’s Republic of China; 3 Zhejiang Provincial Center for Disease Control and Prevention, Hangzhou City, Zhejiang Province, People’s Republic of China; 4 Wenzhou Municipal Center for Disease Control and Prevention, Wenzhou City, Zhejiang Province, People’s Republic of China

## Abstract

**Background:**

A prolonged measles epidemic occurred in Wenzhou City, China after a nationwide measles mass immunization campaign (MMIC) in 2010. We conducted an investigation to identify factors contributing to this epidemic and to provide evidence-based recommendations for measles elimination strategies in China.

**Methods:**

Measles was diagnosed using the national standard case-definitions. We estimated the population vaccination coverage based on the proportion of measles patients that had been vaccinated. In a case–control investigation, all measles patients who received treatment in The Second Affiliated Hospital of Wenzhou Medical College (Hospital S) during November 1 to December 31, 2010 served as cases; controls were randomly selected among all other patients who received treatment in Hospital S during the same time period, frequency matched by month of hospital visit. We reviewed medical records of case- and control-patients to compare their exposure history at Hospital S and to its intravenous rehydration room (IV room) during the incubation period (7–21 days before their illness onset).

**Results:**

The attack rate of measles in Wenzhou City was 3.3/100,000 during September 1, 2010 to January 11, 2011. Children aged 8-11 m had the highest attack rate (171/100,000) of all age groups. In children not age-eligible for the MMIC but should have been routinely vaccinated after the MMIC, the vaccination rate was only 52%. In the case–control investigation, 60% (25/42) of case-patients compared with 21% (35/168) of control-patients had visited Hospital S (adjusted OR_M-H_ = 5.5, 95% CI = 2.7–11). Among unvaccinated children who had received treatment in Hospital S, 84% (21/25) of case-patients compared 38% (11/29) of control-patients had visited the IV room (adjusted OR_M-H_ = 9.2, 95% CI = 1.5–59).

**Conclusion:**

Relaxed routine measles vaccination among children after the MMIC was the main factor responsible for this epidemic. Exposure in the IV room at Hospital S facilitated the epidemic. To reach the goal of measles elimination, the Chinese public health authorities should make greater efforts to improve timely routine measles vaccination, and to reduce nosocomial transmission.

## Background

In China, measles vaccination started in 1978. The original schedule was to have 1 dose of measles vaccine administered to infants at 8 months of age [1]. The schedule was amended in 1985 to give children 2 doses of the measles vaccine: The first dose at the age of 8 months; the second dose at the age of 7 years. In 2006, the age of administration of the second dose was changed from 7 years to 18 months [2].

More than 30 years of measles vaccination in China has greatly reduced measles incidence. In 2005, the Ministry of Health of China pledged to join the global effort to eliminate measles by 2012 (i.e., to reduce measles incidence to below 1/1,000,000, excluding imported cases) [3]. An important strategy recommended by the World Health Organization for achieving this goal is the measles mass immunization campaign (MMIC). Many countries have experienced success with this strategy [4]. During Fall 2010, China implemented a nationwide MMIC designed to administer one dose of catch-up measles vaccine to all age-eligible children free of charge, regardless of their vaccination or disease history.

In eastern China’s Zhejiang Province, the MMIC targeted all children aged 8 m to 4y (born between September 1, 2005 and December 31, 2009). Overall, 2,421,830 children (97% of the targeted children) reportedly were vaccinated in the province during the MMIC, based on the administrative records. Of the 11 cities in the province, Wenzhou City is the most populous. In recent years, Wenzhou City has experienced high measles incidence rates. For example, during January to June in 2005, a measles epidemic occurred in Wenzhou City, with an incidence rate of 30/100,000 [5]; in 2008, the measles incidence rate in one district in the city reached 286/100,000 [6]. During the MMIC in 2010, 443,341 children (98% of the targeted children) reportedly were vaccinated in Wenzhou City, based on the administrative data.

However, soon after the MMIC, surveillance data detected a measles epidemic in the city. The number of reported measles cases during October to December, 2010 was 10 times as high as that during the same period in 2009. We investigated this post-MMIC measles epidemic in Wenzhou City to identify factors contributing to this epidemic and risk factors for measles transmission, and to provide evidence-based recommendations for measles elimination strategies in China.

## Methods

### Data collection

In China, all measles cases are notifiable through the Measles Surveillance System, which is part of the internet-based China Information System for Disease Control and Prevention [7]. Measles cases are diagnosed and classified based on the standard case definitions in the Chinese Measles Surveillance Program [8]. Briefly, a suspected measles case is defined as an illness suspected to be measles infection by a physician, or one with all of the following clinical presentations: 1) fever; 2) maculopapular rash; and 3) cough, coryza or conjunctivitis. A laboratory-confirmed case is a suspected case with positive measles-specific IgM antibodies. A clinically diagnosed case is a suspected case without serological testing, but which during the incubation period was epidemiologically linked to a laboratory-confirmed case. In this investigation, we used the data for all laboratory-confirmed or clinically diagnosed measles cases with illness onset from September 1, 2010 to January 11, 2011 in a permanent or temporary Wenzhou City resident.

### Estimation of measles vaccination coverage for children aged < 15y

We collected the data on vaccination history for measles cases aged 8 m to 15y available through the Measles Surveillance System, and used the following formula to estimate the measles vaccination coverage [9]:

$$\mathrm{PPV}=\frac{\mathrm{PCV}}{1-\left(1-\mathrm{PCV}\right)\times \mathrm{VE}},$$

Where: PPV = Proportion of population vaccinated;

PCV = Proportion of cases vaccinated; and

VE = Vaccine efficacy.

In estimating the PPV, we assumed the efficacy of a single-dose measles vaccine to be 95% [10,11]. We also assumed that the vaccination rate in case-patients with unknown vaccination history was the same as in case-patients with known vaccination history. We classified all case-patients born after January 1, 1996 into 4 age groups, consistent with the Chinese Measles Vaccination Guidelines [12]: Age Group A: Infants aged 0–7 m at the MMIC (born during January 1-November 9, 2010) and were not age-eligible for the campaign, who subsequently contracted measles under age 8 m; these children never became eligible for routine vaccination before their illness onset; Age Group B: Infants aged 0–7 m at the MMIC (born during January 1-November 9, 2010) and were not age eligible for the campaign, who subsequently contracted measles at age ≥8 m; these children should have received post-MMIC routine vaccination before their illness onset; Age Group C: Children aged 13-64 m at the time of this investigation (born during September 1, 2005-December 31, 2009); these children should have been the target of the MMIC; and Age Group D: Children aged 65 m-15y at the time of this investigation (born during January 1, 1996-August 31, 2005); these children were not the target of the MMIC.

### Case–control study

Descriptive analysis revealed that 58% (78/135) of the measles case-patients aged <12 m reportedly had visited a hospital during the incubation period (7–21 days before their illness onset), and 68% (53/78) of those hospital visits occurred in The Second Affiliated Hospital of Wenzhou Medical College (Hospital S). On-site inspection showed that all pediatric patients, regardless of their illnesses, shared the same intravenous rehydration room (IV room) during treatment. We therefore hypothesized that Hospital S and its IV room played an important role in this epidemic. To test this hypothesis we conducted a case–control study in Hospital S. Measles patients aged <12 m who received treatments in Hospital S during November 1 to December 31 served as cases. We randomly selected controls among all other pediatric patients aged <12 m that visited Hospital S during the same time period, frequency matched by month of hospital visit on a 1:4 case-to-control ratio. We reviewed the medical records of case- and control-patients to collect information about their exposure history to Hospital S, especially to its IV room. For case-patients, the exposures of interest were during 7–21 days before their illness onset, whereas for control-patients, 7–21 days before their matched dates of consultation at Hospital S. We collected the vaccination history data of the case- and control-patients from the information system of the provincial Expand Program on Immunization (EPI).

### Ethical considerations

This investigation was part of the response to a public health emergency event and was determined to be exempt from institutional review board review by the Chinese Center for Disease Control and Prevention. All data were kept confidential without patient identifiers.

## Results

From September 1, 2010 to January 13, 2011, 232 suspected measles cases were reported, of which 209 were either laboratory confirmed (n = 127) or clinically diagnosed (n = 82) cases. The estimated attack rate (based on laboratory-confirmed or clinically diagnosed cases combined) was 3.3/100,000, which was about 4 times as high as the overall attack rate in Zhejiang Province (0.83/100,000). The median age of these measles cases was 1y (range: 2 m to 47y). Children aged 8–11 m had the highest attack rate (171/100,000), followed by children aged 0–7 m (Table 1). Measles cases were reported from all 11 districts and counties of Wenzhou City; 59% of the cases were reported from the 3 central urban districts where Hospital S, the largest pediatric hospital in Wenzhou City, is located.

**Table 1:** Attack rate of measles, by age: Wenzhou City, China, September 1, 2010 to January 13, 2011

**Age group**	**# Cases**	**Attack rate (/100,000)**
0-7 m	67	84
8-11 m	68	171
1-5y	21	3.5
6-10y	6	0.90
11-15y	1	0.15
16-47y	46	1.1
Total	209	3.3

**Figure 1:**
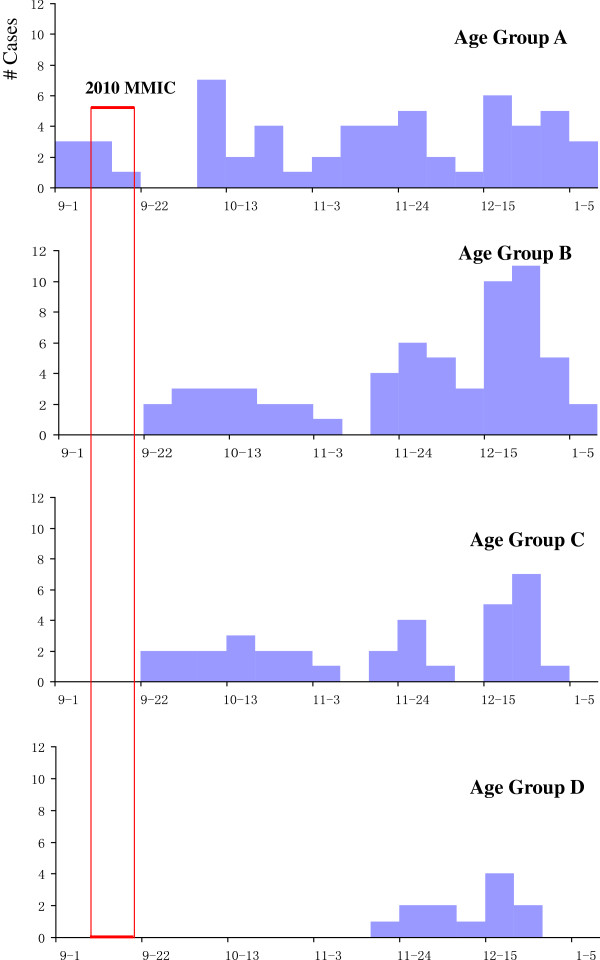
**Age-specific epidemic curves of measles infections: Wenzhou City, China, September 1, 2010 to January 13, 2011.** Age Group **A**: Children aged <8 m at MMIC (born Jan. 1-Nov. 9, 2010), who had measles at <8 m (i.e., never became age-eligible for routine vaccination before illness onset). Age Group **B**: Children aged <8 m at MMIC (born Jan. 1-Nov. 9, 2010), who had measles at > =8 m (i.e., should have received post-MMIC routine vaccination before illness onset). Age Group **C**: Children born Sep. 1, 2005-Dec. 31, 2009: Eligible for MMIC (aged 13-64 m at this investigation). Age Group **D**: Children born during January 1, 1996-August 31, 2005, not the target children of MMIC.

The age-specific epidemic curves showed that, for children not eligible for the MMIC and never became age-eligible for the routine vaccination before their illness onset (Age Group A), cases occurred continuously. For children not age-eligible for the MMIC but should have been routinely vaccinated after the MMIC (Age Group B), the number of cases increased quickly after the MMIC was completed. Among children eligible for the MMIC (Age Group C), cases occurred sporadically until mid-December, 2011, when the case count started to increase. Among older children who were not targeted by the MMIC (Age Group D), the number of cases started to increase after November, 2011 (Figure 1).

The estimated vaccination coverage in none of the age groups exceeded 85%, with the exception of the older children who were not the target of the MMIC (Age Group D). Specifically, for children not age-eligible for the MMIC but should have been routinely vaccinated after the MMIC (Age Group B), the estimated vaccination coverage was only 52%, far below the national target of 95% for measles elimination. Even for the children who were the target of the MMIC (Age Group C), the estimated vaccination rate was only 85%, substantially lower than the national target (Table 2).

**Table 2:** Estimated measles vaccination coverage for children aged 8 m-15y, by age group classified in relation to the nationwide measles mass immunization Campaign: Wenzhou City, China, September 1, 2010 to January 13, 2011*

**Age group#**	**# Cases**	**% Cases vaccinated**	**% Population vaccinated**
A	39	-	-
B	58	5.2%	52%
C	32	21%	85%
D	12	56%	96%

^*^ Assuming vaccine efficacy to be 95%. The vaccination rate in case-patients with unknown vaccination history was the same as in case-patients with known vaccination history.

# A: Infants aged 0-7 m at the MMIC (born during January 1-November 9, 2010) and were not age-eligible for the campaign, who subsequently contracted measles under age 8 m; B: Infants aged 0-7 m at the MMIC (born during January 1-November 9, 2010) and were not age eligible for the campaign, who subsequently contracted measles at age ≥8 m; C: Children aged 13-64 m at the time of this investigation (born during September 1, 2005-December 31, 2009); D: Children aged 65 m-15y at the time of this investigation (born during January 1, 1996-August 31, 2005).

Analysis of the case–control data, which involved 42 cases and 168 controls, showed that the measles vaccine was highly protective (OR_M-H_ = 0.026, 95% CI: 0.0028-0.24) in children aged 8-11 m; the vaccine effectiveness based on this odds ratio (9) was estimated to be 97.4% (95% CI: 76% to 99.7%). Additionally, visiting Hospital S was associated with more than a 5-fold increase in the odds of contracting measles (OR_M-H_ = 5.5, 95% CI: 2.7–11). Children aged 8–12 m were expectedly at higher odds of contracting measles than children aged <8 m (OR_M-H_ = 3.0, 95% CI: 1.5–6.3). When we further examined the exposures of the patients who had visited Hospital S 7–21 days before their illness onset, visiting the IV room was associated with a 7-fold increase in the odds of measles infection (OR_M-H_ = 7.2, 95% CI: 1.9–27) (Table 3). In an analysis of unvaccinated children who had visited Hospital S 7–21 days before their illness onset, stratified by children’s age, we found that IV-room visit was associated with a 9-fold increase in the adjusted odds of measles infection (adjusted OR_M-H_ = 9.2, 95% CI: 1.5–59) (Table 4).

**Table 3:** Univariate analysis of factors for measles infection among children aged ≤1y: Wenzhou City, China, November-December, 2010

**Factors**	**Number**		**Proportion (%)**		OR*_M-H_	**95% CI**
	**Case *n*** **= 42**	**Control *n*** **= 168**	**Case**	**Control**		
**Measles vaccination history**						
Vaccinated	1	38	6.3	49	0.026	0.0028–0.24
Not vaccinated	29	38	94	51	Ref	
**Visit to Hospital S 7-21d before onset**						
*Visited Hospital S*	25	35	60	21	5.5^§^	2.7–11
Visited IV room	21	16	84	46	7.2^¶^	1.9–27
Did not visit IV room	4	19	16	54	Ref	
*Did not visit Hospital S*	17	133	40	79	Ref	
**Age**						
8-12 m	30	76	71	45	3.0	1.5–6.3
<8 m	12	92	29	54	Ref	

* Adjusted for month of visit to Hospital S, using the Mantel-Hanszel method.

§ Compared with children who did not visit Hospital S.

¶ Compared with children who did not visit IV room.

**Table 4:** Association between visiting IV room in Hospital S and measles infection in children under 1 year of age, stratified by age group: Wenzhou City, China, November - December, 2010

	**Age of children**						
**Visited IV room**	**<8 m (n = 26)**			**8-12 m (n = 28)**			**Adjusted OR**_**M-H**_^**§ **^**(95%CI)**
	**Cases**	**Controls**	**OR**_M-H_* (95%CI)	**Cases**	**Controls**	**OR**_M-H_* (95%CI)	
+	5	5	18(1.1–292)	16	6	5.7(0.50–67)	9.2(1.5–59)
-	1	15	Ref	3	3	Ref	Ref

*Adjusted for month of visit to Hospital S, using the Mantel-Hanszel method.

§Adjusted for month of visit and age of children.

An on-site inspection of Hospital S revealed that the hospital’s pediatric clinic had several physician offices. All pediatric patients visiting these physician offices shared the same IV room during treatment. The IV room had an area of about 500 m^2^, with approximately 400 chairs in the room for waiting patients. According to the hospital’s record, 1600–1700 bags or bottles of IV fluid were administered in this room daily. Typically a child was accompanied by two adults; hence 4800–5100 persons are crammed in the IV room on a daily basis, subjecting the children and accompanying adults to the risk of infection by various pathogens. The surfaces and floor of the IV room reportedly were cleaned and disinfected once every morning before the room was opened, but were not disinfected during the day.

## Discussion

A prolonged measles epidemic occurred in Wenzhou City during October 2010 to January 2011 after the nationwide MMIC. Our investigation showed that, in age group A and age group C, the estimated vaccination coverage did not reach the national target of 95%; children not age-eligible for the MMIC but who should have been routinely vaccinated after the MMIC had especially low vaccination coverage at only 52%, suggesting a post-MMIC relaxation of the routine vaccination program; this age group experienced a sharp increase in measles cases after the MMIC, which likely was the main factor responsible for this epidemic.

Measles is highly contagious. Its main modes transmission included droplet transmission, direct contact with nasal or throat secretions of infected persons, and fomite transmission. A patient is infectious from 1 day before the prodromal period (usually about 4 days before rash onset) to 4 days after the appearance of rash [13].

The most important strategy to control and eliminate measles is to improve the coverage rate of the measles vaccine [4], which has an excellent efficacy [10,11,14]. However, nosocomial transmission can play an important role in measles epidemics [15,16]. Measles is difficult to diagnose before rash onset, easily leading to diagnostic errors and hence nosocomial transmission when the care-seeking measles patients are mixed with other patients in hospitals or clinics [15,16]. In China, few stand-alone private clinics currently exist; outpatient visits usually occur in the outpatient building adjacent to the main hospital building. Those outpatient buildings are usually extremely crowded. Furthermore, Chinese patients receive IV injections much more frequently than patients elsewhere [17,18]; consequently, the IV rooms are typically crammed with patients, serving as a perfect venue for the transmission of various pathogens [19].

Our investigation had at least three limitations. First, we estimated the measles vaccination coverage based on the proportion of cases vaccinated. In the calculation we assumed that the efficacy of the vaccine was 95%, which may not be valid in all situations. A better way to estimate vaccination coverage is to conduct a population sample survey, but such a survey would have been resource- and labor-intensive, and exceeded our capacity. Second, some of our data were obtained from various reporting and registration systems (e.g., the Measles Surveillance System, the EPI information system, and the hospital medical records), which may be incomplete. Third, our case–control investigation was hospital-based rather than population-based, which might have produced selection bias.

## Conclusions

Relaxation of routine measles vaccination after the nationwide MMIC was the main factor contributing to this measles epidemic. Transmission in the IV room of Hospital S facilitated this epidemic. To reach the goal of measles elimination, the Chinese public health authority should make greater efforts to identify the unvaccinated populations, and to expand routine measles vaccination to these populations. They should also improve the environment and management of hospitals to reduce nosocomial transmission of infectious diseases by screening and triaging of febrile patients, distributing face masks to patients at outpatient buildings, and providing convenient hand-washing facilities for patients.

## Competing interests

The authors declare that they have no competing interests.

## Authors’ contributions

JG and EC contributed to study design, data collection, cleaning and analysis. JG prepared the original manuscript. ZW, JS and HH participated in the implementation of the study and contributed to data collection. HM and B-PZ contributed to study design, data analysis and interpretation, and preparation of the final manuscript. GZ contributed to study design, interpretation, and preparation of the final manuscript. All authors had access to all data and contributed to the final draft of the paper. All authors read and approved the final manuscript.

## Pre-publication history

The pre-publication history for this paper can be accessed here:

http://www.biomedcentral.com/1471-2334/13/139/prepub
